# Excessive aggregation of membrane proteins in the Martini model

**DOI:** 10.1371/journal.pone.0187936

**Published:** 2017-11-13

**Authors:** Matti Javanainen, Hector Martinez-Seara, Ilpo Vattulainen

**Affiliations:** 1 Laboratory of Physics, Tampere University of Technology, Tampere, Finland; 2 Department of Physics, University of Helsinki, Helsinki, Finland; 3 Institute of Organic Chemistry and Biochemistry, Czech Academy of Sciences, Prague, Czech Republic; 4 Memphys - Center for Biomembrane Physics; Danish Cancer Society Research Center, DENMARK

## Abstract

The coarse-grained Martini model is employed extensively to study membrane protein oligomerization. While this approach is exceptionally promising given its computational efficiency, it is alarming that a significant fraction of these studies demonstrate unrealistic protein clusters, whose formation is essentially an irreversible process. This suggests that the protein–protein interactions are exaggerated in the Martini model. If this held true, then it would limit the applicability of Martini to study multi-protein complexes, as the rapidly clustering proteins would not be able to properly sample the correct dimerization conformations. In this work we first demonstrate the excessive protein aggregation by comparing the dimerization free energies of helical transmembrane peptides obtained with the Martini model to those determined from FRET experiments. Second, we show that the predictions provided by the Martini model for the structures of transmembrane domain dimers are in poor agreement with the corresponding structures resolved using NMR. Next, we demonstrate that the first issue can be overcome by slightly scaling down the Martini protein–protein interactions in a manner, which does not interfere with the other Martini interaction parameters. By preventing excessive, irreversible, and non-selective aggregation of membrane proteins, this approach renders the consideration of lateral dynamics and protein–lipid interactions in crowded membranes by the Martini model more realistic. However, this adjusted model does not lead to an improvement in the predicted dimer structures. This implicates that the poor agreement between the Martini model and NMR structures cannot be cured by simply uniformly reducing the interactions between all protein beads. Instead, a careful amino-acid specific adjustment of the protein–protein interactions is likely required.

## Introduction

Aggregation of proteins has severe implications for health. For instance, G protein-coupled receptors (GPCRs) form complex functional oligomers that act as drug targets in membranes [1, 2]. Also, the aggregation of misfolded proteins is considered to be the cause of numerous neurodegenerative conditions such as Alzheimer’s disease [3]. Without doubt, there is a need to understand how and why proteins arrange themselves into oligomers.

The molecular dynamics (MD) simulation technique has been applied quite extensively to study the oligomerization and aggregation of membrane proteins including GPCRs [4–11] (see also the extensive review by Periole [12]) as well as members of other membrane protein classes [13, 14]. Quite a few studies have also focused on the lateral diffusion dynamics in protein-rich membranes [15–18]. The most ambitious MD simulations have studied the assembly of respiratory chain supercomplexes [19] and the complete influenza A virion, whose surface is crowded with proteins [20].

All of these studies have used the coarse-grained (CG) Martini model [21–23] or its relatives [24] to probe time and length scales that are beyond the reach of fully atomistic simulations. Especially the Martini model has become very popular in the biomolecular simulation community due to its large library of molecule types, efficiency, as well as availability of high-quality simulation and analysis tools [25–30]. Martini has been parametrized [21] to contain a fairly limited set of bead types, each describing a group of 2–4 heavy atoms. This guarantees speed, simplicity, and transferability, while it also provides an adequate level of chemical specificity for many applications. Notably, the Lennard-Jones (LJ) interactions between the 18 bead types are described by a total of 9 interaction levels with the interaction strength *ϵ* ranging from 2.0 to 5.6 kJ/mol, and the interaction distance parameter *σ* having either a value of 0.62 (super repulsive type) or 0.47 nm (all other types). Additional “small” beads used in rings are described with *σ* = 0.43 nm together with *ϵ* that is reduced by 25% of the normal values. The original Martini model [21] also contains a limited set of bonded parameters. The masses are set to either 72 amu or 45 amu (“small types”), and partial charges exist as multiples of 1 e.

The bead types used in the Martini model were selected to provide liquid state conditions at room temperature and also to reproduce the partitioning coefficients of selected solutes between water and organic solvents [21]. Similarly, the Martini protein model [22] was parametrized using the same bead types, though the originally limited bonded interactions were expanded to provide correct geometries for the different amino acids and protein secondary structures. The performance of the parametrization was assessed based on the partitioning of amino acids between water and cyclohexane [22]. The protein force field was later fine-tuned based on the partitioning free energy between water and decane [23]. Even though numerous other checks were performed at both stages of the parametrization—including the evaluation of some amino acid dimerization free energies—[22, 23] the strength of protein–protein interactions—which cannot be predicted from those of individual amino acids—was actually not validated against experimental data [31].

This issue was brought up recently as Stark *et al*. [32] showed that interactions between water-soluble proteins in the Martini model are pronounced, leading to precipitation at concentrations much below the solubility limit [23]. By using the second osmotic virial coefficient as the target experimental value, Stark *et al*. [32] showed that it can be reproduced if the Lennard-Jones interaction strength between protein beads is drastically weakened [32]. In the same spirit, dimerization free energies calculated for membrane proteins and peptides indicate a very strong tendency for dimerization [11, 12, 33–38] as exemplified by the dimerization free energies of about −150 kJ/mol and −160 kJ/mol reported for outer membrane protein F (OmpF) [38] and *κ*-opioid receptor (KOPr) [11], respectively. Even though free energies cannot be directly linked to dimerization kinetics, it is obvious that such strong affinities indicate irreversible binding.

Förster resonance energy transfer (FRET) enables the calculation of dimerization free energies in simple model liposomes [39] as well as in liposomes constructed from mammalian plasma membrane extracts [40]. FRET provides association constants, which can be converted to dimerization free energy values allowing direct comparison of simulation with experiment. Still, it must be kept in mind that the values provided by FRET are obtained indirectly and are certainly not free of uncertainties. Notably, a dimer is defined as a state in which the fluorescent labels attached to the monomers—residing outside the membrane—are reasonably close to each other. Despite these limitations, FRET can be considered to be the most reasonable technique to provide dimerization free energies of membrane proteins in lipid membranes.

Consistent experimental dimerization data exist for certain protein types, allowing a straightforward comparison to simulations. The transmembrane (TM) domains of proteins in the receptor tyrosine kinase (RTK) class are a prime example. To begin with, FRET reported a value of −10.5 kJ/mol for the TM segment of an ErbB1 receptor in a DLPC bilayer [41]. Meanwhile, a Martini simulation of this dimer in a DPPC bilayer provided a much larger value of −25.5 kJ/mol [42], and a recent study with a Martini-derived force field on a ErbB1 homology model in a DPPC bilayer reported a value of −38 ± 3 kJ/mol [43]. The dimerization free energy of another RTK TM segment, the Ephrin type-A receptor 1 (EphA1), was measured by FRET to be −15.4 kJ/mol in a DMPC bilayer [44]. Meanwhile, Martini simulation provided a substantially larger value of −60 kJ/mol in a DPPC bilayer [34]. Even though simulation data do not exist for the fibroblast growth factor receptor 3 (FGRF3) that is also a member of the RTK class, experiments in POPC liposomes reported dimerization free energies between −11.3 kJ/mol and −12.6 kJ/mol even when mutations are present [45–48]. Concluding, a number of results for the RTK TM domains point to dimerization free energies in the ballpark of −10 to −15 kJ/mol, while Martini simulations consistently provide too stable dimers with dimerization free energies ranging between −25 and −60 kJ/mol. While there is still room for speculation since the computational setups do not usually match the experimental ones, the significant discrepancy between experiment and Martini model predictions gives rise to concern.

The disagreement is not always this evident. For example, FRET experiments reported Glycophorin A (GpA) dimerization free energies in the range of −(13–17) kJ/mol in various cell membranes [40, 49], while Martini simulations in model bilayers provided much larger values of −26 kJ/mol, −35 kJ/mol, and −40 kJ/mol [50–52]. However, experiments performed using a steric trap obtained values of −50.6 kJ/mol for GpA in a POPC membrane [53], while values of −31.6 kJ/mol [54] and −31.4 kJ/mol [55] were measured by a GALLEX assay in *E. coli* inner membranes.

Similarly, the dimerization free energy of the WALP23 peptide was calculated from a Martini simulation in a DOPC membrane to be −20 kJ/mol [56], while values of −12.2 ± 2 kJ/mol [57] and −15 kJ/mol [58] were measured in DOPC and DLiPC bilayers, respectively. These values can also be compared to those measured for the structurally similar (AALALAA)_3_ peptide, for which a value of −12.7 ± 0.4 kJ/mol was obtained with FRET [59]. On the other hand, the dimerization free energy of these (AALALAA)_3_ peptides was seen to be well reproduced with Martini but not with an atomistic force field [31]. Interestingly, a study comparing Martini with several atomistic force fields indicates that the atomistic force fields usually display lower dimerization free energies and some even underestimate them in comparison to experiments. [31]

The large scatter of the above listed experimental values that depend on the experimental technique and setup complicates comparison to simulations. However, most of the listed evidence points towards Martini predicting too strong dimerization. This is a potentially severe issue, since too large dimerization free energies lead to several problems: 1. Extracting information on the lateral diffusion in realistic crowded-like conditions is compromised due to pronounced aggregation, which unrealistically confines the diffusion of proteins as well as lipids. 2. Resolving favorable lipid–protein interactions in membranes with multiple proteins is undermined as the affinity of proteins for the lipid binding sites is higher than that of lipids. 3. Studying protein–protein dimerization interfaces becomes incomplete even in exceedingly long Martini simulations as proteins aggregate upon first contact and do not properly sample all possible configurations.

In this work, we show that the dimerization free energies obtained using the non-polarizable and polarizable variants of Martini for TM segments of RTKs are substantially larger than those measured in FRET experiments. We also show that the agreement between the simulation-based structures of spontaneously formed TM segment dimers and the structures resolved using NMR leaves a lot of room for improvement. We attempt to improve the situation of the non-polarizable Martini model by downscaling the protein–protein interactions without affecting other carefully parametrized parts of the Martini model. We find that the modification suggested by Stark *et al*. [32] (downscaling of the protein–protein LJ *ϵ* parameter by 60%) is too drastic to resolve the excessive binding issue found with membrane proteins. On the other hand, applying this modification to only parts of the protein residing in the water phase does not reduce the dimerization energies sufficiently. Meanwhile, when we apply a milder (10%) and uniform reduction in all protein–protein interactions, we find the dimerization free energies of TM segments to closely match experimental data, allowing for studies on membrane dynamics and protein–lipid interactions. However, the simulation structures of spontaneously formed dimers of transmembrane peptides are not improved, which calls for a more careful amino acid specific adjustment of the interaction levels.

## Materials and methods

### Umbrella sampling simulations

We consider RTK TM domain dimerization as a representative case for studying protein–protein interaction. This choice is made for two reasons. First, a large amount of experimental data exists for this protein class. Many TM domain dimer structures have been resolved by NMR and hence this information can be exploited to validate that the most favorable dimerization interface is studied in the simulations. Second, despite their flexibility, the convergence is more likely with TM domains formed by single helices rather than with more complex proteins, such as those composed of multiple helices or beta barrels.

**Fig 1:**
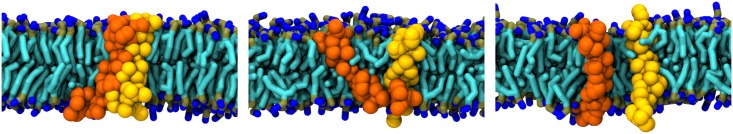
Selected snapshots of transmembrane domain dimerization. Examples of conformations at different COM–COM distances (from top to the bottom: 1.2, 2.0, and 2.4 nm) from the umbrella sampling simulations (COM stands for the center of mass). Here, the U-0.1 scaling method is employed (see below). Chains of lipids (DLPC) are shown in cyan, phosphate beads in brown, and choline beads in blue. Peptides (EphA1) are shown in yellow and orange. Water, antifreeze particles, and ions have been omitted from the images.

The EphA1 dimer (PDB [60] entry 2K1L [61]) was embedded [27] in a lipid bilayer comprised of 400 DLPC molecules (indistinguishable from DMPC in Martini). The system was hydrated by 12.5 water beads (corresponding to 50 water molecules) per lipid out of which 10% were presented by the antifreeze type water beads. A physiological salt concentration of 150 mM of NaCl was included to the system (in addition to counter ions) to match the setup employed in experiments [44]. See Fig 1 for selected snapshots of the system.

In addition, a dimer of ErbB1 TM domains (PDB [60] entry 2M0B [62]) was embedded into an identical DLPC bilayer. The only difference to the EphA1 system described above, in addition to the protein, was that the NaCl concentration was higher at 500 mM in order to match the experimental setup [41].

The systems were first simulated for 50 ns with restraints on the dimer, so that the membrane was able to adapt to the newly included dimer. The restraints were then released and the system was simulated for another 50 ns. Next, using the lateral distance between the centers of mass (COMs) of the TM domains as the reaction coordinate, the peptides were pulled apart to generate initial structures for umbrella sampling windows. In these initial structures, the COM distance varied between 0.6 and 5.0 nm with a spacing of 0.2 nm.

The non-polarizable Martini model version 2.2 was employed [23] in the simulations together with the suggested “New-RF” simulation parameter set. [63] More concretely, the reaction field electrostatics and Lennard-Jones potentials were shifted to zero at the cut-off distance of 1.1 nm. A dielectric constant of 15 was employed up to the cut-off length, after which it was given a value of infinity. The Verlet cut-off scheme was employed with parameters defined by Gromacs. Temperatures of the dimer, the lipids, and the solvent were separately kept constant at 303 K with the stochastic velocity rescaling thermostat [64], while pressure was maintained semi-isotropically at 1 bar using the Parrinello–Rahman barostat [65] with a time constant of 12 ps and a compressibility of 3 × 10^−4^ bar^−1^. Additionally, as a reference we repeated the umbrella sampling simulations for the EphA1 dimer with the older set of suggested simulation parameters, referred to as “Common” (for details, see Ref. [63]), as well as using the recently updated polarizable parameters [66, 67], combined with the “New-RF” simulation parameters (with a dielectric constant of 2.5) [63]. In all simulations, the elastic network [68] with default options, *i.e.* a force constant of 500 kJ mol^−1^ nm^−2^ and an upper cut-off of 0.9 nm were employed for the TM domains.

For the umbrella sampling, the lateral distance of the COMs of the two TM segments was restrained with a harmonic spring with a constant of 400 kJ mol^−1^ nm^−2^, which provided a suitable overlap in the umbrella histograms. The selected umbrella windows (see Results) were each simulated for 10 μs with a time step of 20 fs, and the first 50 ns of each window was discarded from analyses. The COM–COM distances were stored every 10 time steps (200 fs). Potential of mean force (PMF) profiles, estimating the free energy profile of dimerization (*G*(*r*)) were extracted using the g_wham tool [69]. The same tool was used to estimate the error bars in the PMF profiles using 100 bootstrap samples. All simulations were performed with the Gromacs version 5.0.x [70].

The dimerization free energy Δ*G*_DIM_ calculated from FRET experiments corresponds to the probability of the TM segments being in any bound state, *i.e.* the depth of the well in the free energy profile cannot be directly compared to experiment. Rather, an average needs to be calculated using [50]

$$\begin{array}{c} \Delta G_{\text{DIM}}=-RT\;\text{ln}\;(K_{\text{a}}), \end{array}$$ (1)

where

$$\begin{array}{c} K_{a}=\pi \int_{0}^{r_{\text{max}}}r\exp(-\frac{G(r)}{RT})\;dr. \end{array}$$ (2)

Here, *K*_a_ is the association constant, *R* the gas constant, *T* the temperature, and *G*(*r*) the free energy change (with respect to the chosen zero-level) at a COM–COM distance *r*. The usual standard state used in FRET experiments [40], 1 nm^2^ per receptor, is also adapted here. The *G*(*r*) profiles are shifted to zero at a COM–COM distace of 2.75 nm. Most profiles have reached a plateau at this arbitrarily chosen distance. The integration limit (*r*_max_) is set to 2.0 nm. The *r*_max_ value is larger than the used cutoff lengths, and the increase of *r*_max_ above 2.0 nm has an insignificant effect on the obtained Δ*G*_DIM_ value. The error in the Δ*G*_DIM_ values is the standard deviation of the individual values extracted from the 100 bootstrapped *G*(*r*) samples.

### Spontaneous dimer formation

We also evaluated the ability of the Martini model to predict the structures of TM domain dimers. For this purpose, we considered TM domains that are known to dimerize [71]. Notably, to this end we chose 1) Glycophorin A dimer (PDB entry 1AFO [72]), 2) integrin *α*IIb*β*3 heterodimer (PDB entry 2K9J [73]), 3) BNIP3 TM domain dimer (PDB entry 2KA1 [74]), 4) T cell receptor signaling module *ζζ* (PDB entry 2HAC [75]), and 5) DAP12 signaling subunit dimer (PDB entry 2L34 [76]). As initial simulation structures, the peptides in the dimer structures were separated by ∼4 nm and coarse-grained using the martinize tool, and embedded in a DLPC bilayer with insane [27]. The bilayers consisted of ∼400 lipids and were hydrated by ∼6000 water beads, out of which 10% was presented with the antifreeze type. About 150 mM of NaCl was added to the water phase together with counter ions.

The assembled systems were first equilibrated for 50 ns with position restraints applied to the protein beads, after which 10 μs simulations for generating initial structures for the production simulations were performed for each system. During these simulations, the distance between the COMs of the peptides was kept at ∼4 nm with a harmonic potential with a spring constant of 400 kJ mol^−1^ nm^−2^. Next, a total of 10 structures were extracted at 1 μs intervals, and employed as independent replicas with different initial structures. The 10 replica simulations were run with both unscaled Martini parameters as well as with the U-0.1 scaling strategy (see below). Therefore, the total number of these unbiased simulations was 5 (dimer structures) × 2 (scaled vs. unscaled) × 10 (replicas). Each of these simulations was run for 40 μs with the “New-RF” simulation parameters (see above), resulting in a total of 4 ms of simulation time.

The quality of the dimeric structures from these unbiased simulations was evaluated from the last 20 μs of simulations using three criteria: root-mean-square deviation (RMSD) of the backbone beads from the coarse-grained NMR structures of the dimer, the deviation of the dimer crossing angles from their values extracted from the coarse-grained NMR structures, and the deviations in the number of inter-peptide contacts (defined with a cut-off of 0.8 nm) between the backbone beads in the coarse-grained NMR structure. The dimer crossing angles were calculated as the mean angles between the principal moments of inertia of the peptides calculated using a single value decomposition of the backbone beads of the residues defining the contact helices (See S1 Table in Supporting Information (SI)).

### Order of oligomerization

We evaluated whether EphA1 and ErbB1 form dimers, as observed in experiments [41, 44], or higher order oligomers. To this end, we began with the structures extracted from the umbrella sampling windows with the lateral protein–protein distance equal to ∼3 nm. We then replicated this system in the membrane plane four-fold (2 × 2). The systems containing a total of 8 monomers were simulated using both unscaled Martini parameters and the U-0.1 scaling strategy (see below) for 40 μs. The “New-RF” simulation parameters were again used (see above). The center of mass trajectories of the peptides were created with g_traj and analyzed with the tool g_clustsize. We used a cut-off of 2.5 nm for the clusters, as the radial distribution functions for the peptide COMs converged to zero at approximately this distance.

### Adjustment of protein–protein interaction levels

We consider two ways to scale down the LJ interactions among proteins in order to lower the dimerization free energies to a level observed in FRET experiments. These two approaches are described below. In Martini, the LJ interaction parameters are not calculated from combination rules but are instead tabulated as explained in the interaction matrix shown in the original Martini publication [21]. Therefore, the *ϵ* parameters considered here refer to the interaction parameters of two protein beads (*i*, *j*, often denoted *ϵ*_*i*,*j*_) instead of the LJ parameter of a single bead.

#### Approach U: Uniform scaling applied to all beads

The Lennard-Jones interactions between all protein beads are here reduced by scaling down *ϵ* with a fixed scaling parameter *α* (*α* = 0.1 for a reduction of 10%, etc.). This method is identical (despite the reversed definition of *α*) to that presented by Stark *et al*. [32] The scaled *ϵ*-parameter *ϵ*_scaled_ calculated from the original value *ϵ*_original_ is

$$\begin{array}{c} \epsilon_{\text{scaled}}=\epsilon_{\text{repulsive}}+\left(1-\alpha \right)(\epsilon_{\text{original}}-\epsilon_{\text{repulsive}}), \end{array}$$ (3)

where *ϵ*_repulsive_ corresponds to the weakest (repulsive) interaction in the Martini model (2 kJ/mol). Notably, in this method the values of *ϵ* are never smaller than 2 kJ/mol, which leads to a smaller spread of *ϵ* values and hence to less chemical specificity upon increasing *α*. Unlike in the original work in the aqueous phase, we scale down the protein–protein interactions involving all bead types, including P4, Qa, and Qd. In this paper, the scaled results using this method will be labeled “U-*α*” with “U” standing for uniform scaling.

#### Approach W: Scaling applied to water-contacting beads

Motivated by the good agreement with experiment based on scaling protein–protein interactions in water-soluble proteins by 60% (*α* = 0.6) [32], in this approach we scale down the protein–protein interactions of only those beads in membrane proteins that are mostly in contact with water (see details below). The interactions among these beads are adjusted following the approach “U” described above. The results obtained using this method will be labeled “W-*α*” with “W” standing for water.

The beads to which the scaling was applied were determined as follows: First, the dimer was simulated for 1 μs, and the contacts between each residue with water and lipids were counted after a 50 ns equilibration period. Second, the sesidues that had more contacts with water than with lipids (defined by a cut-off of 0.6 nm) were assigned new particle types, whose mutual interactions were scaled as in the “U” approach. The interactions between scaled and unscaled protein beads remained as in standard Martini.

## Results and discussion

### Dimerization free energies suggested by Martini are excessive

**Fig 2:**
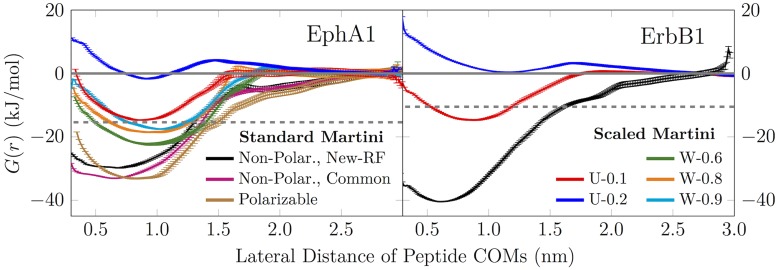
Dimerization free energy profiles of transmembrane domains. Free energy profiles of EphA1 and ErbB1 TM domain dimerization using either unmodified Martini or the “U” and “W” scaling strategies. The solid gray line at a value of zero is to guide the eye. The dashed gray line shows the experimental value for Δ*G*_DIM_. Note that the common legend is split between the two panels.

Using the EphA1 and ErbB1 dimer systems, we evaluated the ability of the Martini model to capture the dimerization free energies of TM domains. Fig 2 depicts the free energy profiles (PMFs) calculated for the EphA1 and the ErbB1 dimer using either the non-polarizable (“Non-Polar.”) or the polarizable (“Pol.”) Martini model. The use of “Common” simulation parameters instead of the “New-RF” ones is indicated by an asterisk (*). All curves have been shifted to realize convergence to zero at a COM–COM distance of 2.75 nm. The values extracted from these profiles using Eqs (2) and (1) are shown in Table 1.

**Table 1:** Transmembrane domain dimerization free energies.

	EphA1	ErbB1
Experimental [41, 44]	−15.4 ± 0.5	−10.5 ± 0.4
Non-Polar. Martini	−29.9 ± 1.0	−39.5 ± 1.0
Non-Polar. Martini*	−32.9 ± 0.5	–
Polar. Martini	−33.5 ± 1.0	–
Scaled U-0.1	−15.2 ± 1.0	−15.3 ± 0.3
Scaled U-0.2	NA	NA
Scaled W-0.6	−23.2 ± 0.8	–
Scaled W-0.8	−19.6 ± 1.4	–
Scaled W-0.9	−18.4 ± 0.4	–
Prev. Studies [34, 42, 43]	−60 ± 2	−25.5 ± 0.6 −38 ± 3

EphA1 and ErbB1 TM domain dimerization free energies Δ*G*_DIM_ (in kJ/mol) calculated from Eqs (2) and (1). In two cases, the dimer does not represent the minimum free energy state and hence Δ*G*_DIM_ values cannot be extracted (NA (not available)). The systems marked with “–” were not simulated. The system simulated with the “Common” parameter set instead of the “New-RF” (see Ref. [63]) setup is denoted with *. Standard and polarizable versions of Martini are denoted by “Non-Polar.” and “Polar.”, respectively.

Using FRET, Artemenko *et al*. [44] measured a dimerization free energy of Δ*G*_DIM_ = −15.4 ± 0.5 kJ/mol for the EphA1 TM domains in DMPC liposomes at 303 K [44], *i.e.* in a setup matching our computational one. They also verified that EphA1 TM segments form dimers and not higher order oligomers. On the other hand, earlier Martini simulations of EphA1 in a DPPC membrane at 323 K provided a drastically different value of −60 ± 2 kJ/mol for the depth of the well in the free energy profile [34].

As shown in Table 1, both the non-polarizable and polarizable Martini models provide values that are drastically higher than the ones obtained from experiments. The non-polarizable Martini provides a value of −29.9 ± 1.0 kJ/mol that is approximately twice the experimental value. Importantly, the choice of simulation parameters (“New-RF” vs. “Common”) has a small effect on the value: the dimerization free energy is slightly larger with the latter parameter set likely due to the longer cutoffs for non-bonded interactions. In addition, the polarizable Martini force field also provides very similar results suggesting that it also suffers from protein–protein overbinding. In all these simulations with an unscaled Martini model (non-polarizable or polarizable), the free energy profiles calculated do not fully reach a plateau even at an inter-protein distance of 3 nm. The protein–protein interactions are so strong that the monomers tilt to maintain any interaction at such large distances, resulting in a monotonous profile.

As to the ErbB1 dimer, FRET measurements by Chen *et al*. [41] in DLPC liposomes (with 500 mM NaCl) for Δ*G*_DIM_ of the TM domains provided −10.5 ± 0.5 kJ/mol. In these experiments, it was again verified that no higher order oligomers were formed. Notably, two independent FRET techniques were employed, and both of them provided almost identical Δ*G*_DIM_ values. A recent metadynamics study using the non-polarizable Martini model, on the other hand, provided a much larger value of Δ*G*_DIM_ = −38 ± 3 kJ/mol in a DPPC bilayer [43]. This value was obtained by a thorough sampling of the two-dimensional free energy surface (COM–COM separation and crossing angle). Additionally, an earlier Monte Carlo study using the Martini model in a DPPC bilayer at 323 K provided a value of Δ*G*_DIM_ = −25.5 ± 0.6 kJ/mol [42].

The value reported by Lelimousin *et al*. [43] (Δ*G*_DIM_ = −38 ± 3 kJ/mol) is in excellent agreement with the value we obtained here with the standard Martini model (Δ*G*_DIM_ = −39.5 ± 1.0 kJ/mol). This suggests that the two-dimensional sampling technique is likely not necessary when the structure of the dimer is readily available from experiments, as is the case with the two TM domains used as examples in this work.

Similarly as for the EphA1 dimer, our free energy profiles converge at unusually large inter-protein distances indicating that the monomers try to remain in contact by tilting toward each other. Indeed, as shown in S1 Fig for the ErbB1 dimer using the non-polarizable Martini (see the SI), the peptides show a systematic tilting at large inter-protein distances.

The experiments for EphA1 and ErbB1 dimerization [41, 44] suggested that neither of these peptides forms higher order oligomers. Using unscaled Martini, we observed that EphA1 rapidly forms two tetramers, while ErbB1 oligomerizes slower but eventually assembles into an octamer. The time evolution of the average size of the oligomers is shown on the left in S3 Fig, and the histogram of the oligomer sizes during the last 10 μs of the simulation on the right in S3 Fig.

### Martini cannot predict dimer structures correctly

We also evaluated the ability of the non-polarizable Martini model to predict the structures of five dimers of TM domains, resolved by NMR (see Methods). This provides an alternate comparison between simulation and experiment to the free energies presented in the previous section.

**Fig 3:**
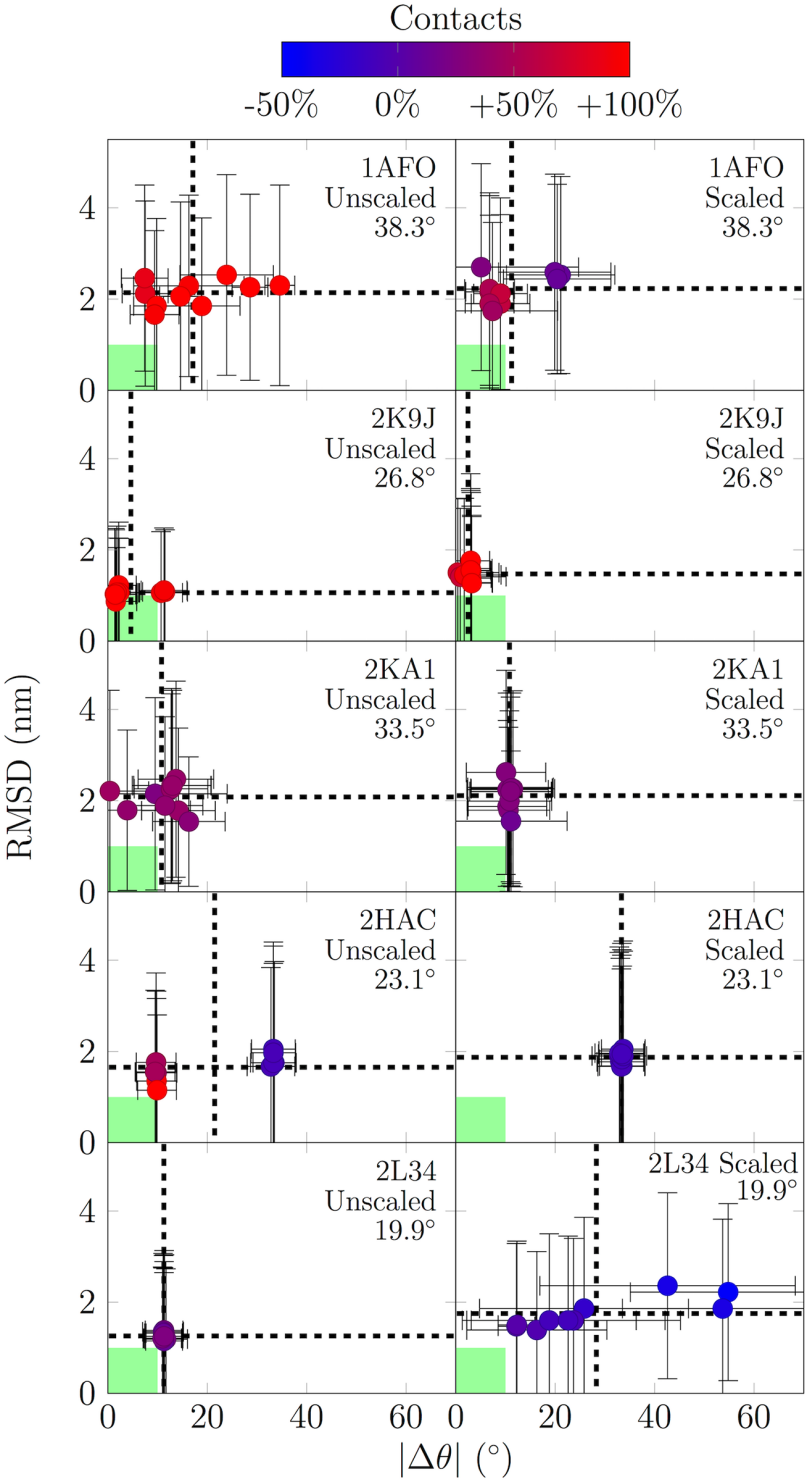
Deviation of the spontaneously formed dimer structures from their NMR structures. Data is shown for both the standard (non-polarizable) Martini and the U-0.1 scaling. The root-mean-square deviation (RMSD) is shown on the *y* axis, and the absolute value of the deviation of the dimer crossing angle on the *x* axis. The coloring shows the deviation in the number of backbone bead contacts (cutoff of 0.8 nm). Therefore, an optimal result would be a purple dot (correct number of contacts) at the origin (RMSD equal to 0 nm and with the correct dimer crossing angle). As a rule of thumb, to guide the eye, the acceptable region (deviations up to 10° and 1 nm considered to be acceptable) is highlighted in green. In each graph, data are shown for 10 replica simulations. Each replica is depicted with a marker with error bars showing standard deviation, while the mean over replicas is highlighted by the dashed lines. The crossing angle of the NMR structure is given for each dimer in degrees. Data are extracted from the last 20 μs of the simulations.

The COM–COM distance of the two peptides is shown in S2 Fig (see the SI), while a careful structural comparison between spontaneously formed dimers and the NMR structures is summarized in Fig 3.

The distances shown in S2 Fig (see the SI) indicate that all observed TM domains form dimers in a few microseconds, as expected. Fig 3 shows that simulations with the standard (non-polarizable) Martini are not able to predict the structures of spontaneously formed dimers correctly. In this figure, the simulation structure coinciding perfectly with the NMR structure would be a purple dot located at the origin. While it is difficult to decide how large deviations from the perfect match are acceptable, we have chosen to highlight in green (Fig 3) a deviation of 10° (crossing angle) and 1 nm (RMSD) that we consider to be reasonable. This choice should help in assessing how well the simulation results agree with experimental data. Importantly, our data clearly show that the number of inter-peptide contacts in the dimer is often severely overestimated for the standard Martini, in line with the excessive dimerization free energies. Interestingly, for the 2L34 dimer, all replicas using the standard Martini fall in the same point in the graph, while other dimers show larger scatter of the replicas indicating a more diverse set of molecular conformations. This might result from the irreversible dimerization of the peptides upon first contact.

**Fig 4:**
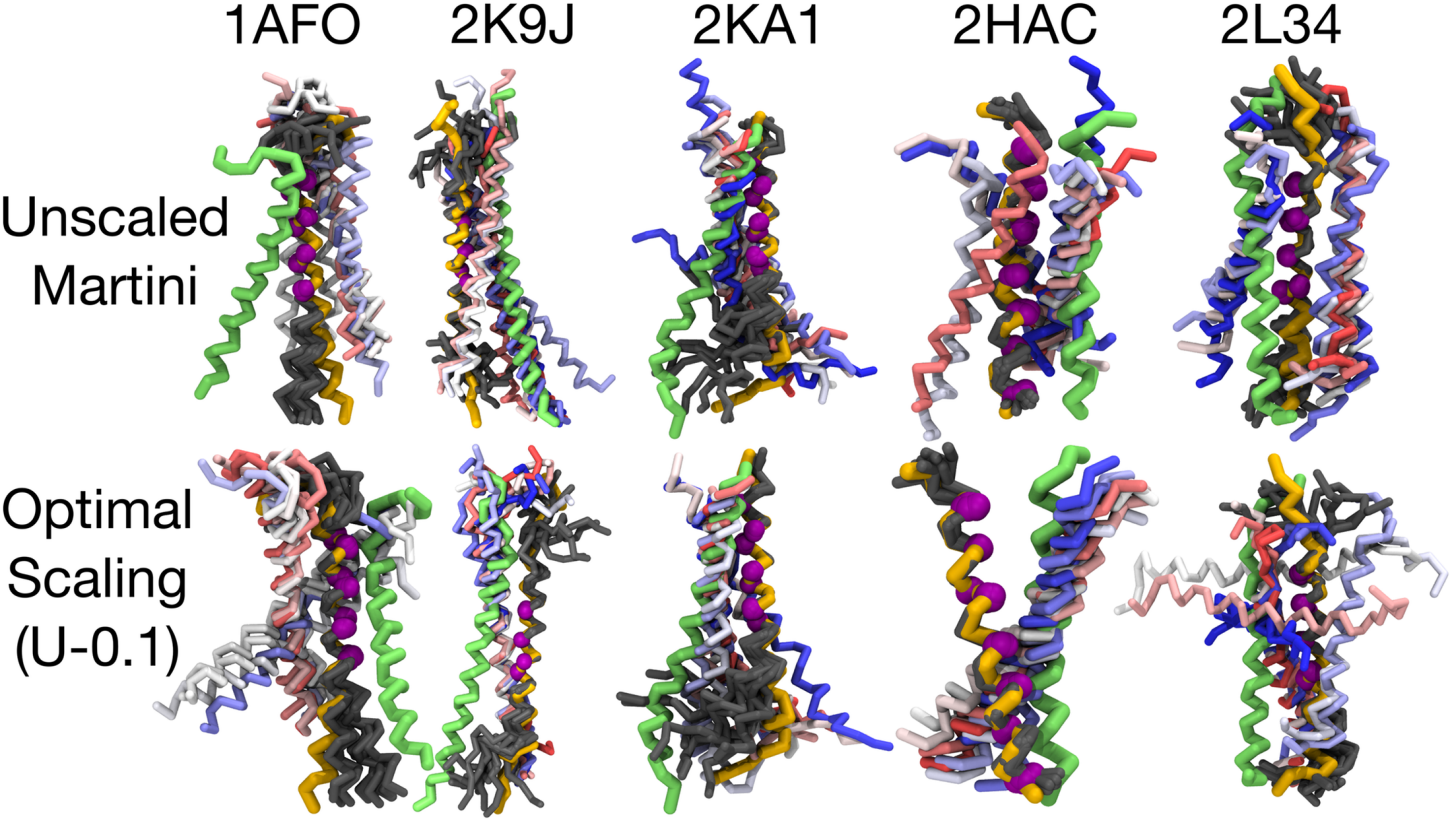
Final structures of the 10 validation replica simulations. Shown here are the structures for both standard (non-polarizable) Martini and the best scaling strategy resolved in this work (U-0.1). The two peptides in the NMR structure are shown in yellow (first peptide) and green (second peptide). The dimerization motif (see Ref. [71]) of the first peptide is additionally highlighted in purple. The dimer structures based on the simulations are depicted in a manner, where the first peptides of the replicas (all shown in gray) are RMSD-aligned at the dimerization motif (purple) on the first peptide of the NMR structure (yellow). The reader should here concentrate on the second peptide structures (not aligned) given by the 10 replica simulations that are depicted by a spectrum of red-white-blue. If there is good agreement between simulation and NMR, the structures of the second peptide (red-white-blue) should coincide with the second peptide in the NMR structure (green).

Still our approach of projecting the dimer structures onto three collective variables does not provide a full picture of the quality of the structures. To provide a more intuitive comparison between simulation and experiment, the final structures of all replicas of the spontaneous dimerization simulations are shown together with the corresponding NMR structures in Fig 4. Here, the structures are aligned to the NMR structure based on an RMSD fit of the dimerization motif of the first (in PDB) peptide, shown in yellow [71]. This motif is highlighted by purple spheres and the obtained fitted replicas are depicted in gray. The other (not RMSD-aligned) peptide from NMR is shown in green. Finally, the position of the not RMSD-aligned peptides from the replicas is shown with colors from a spectrum of red-white-blue. This representation allows a straightforward evaluation of the spontaneously formed dimers by simply comparing the location of the second peptide observed in simulations (red-white-blue) with its location in the NMR structure (green).

It is evident that in most cases the simulations are unable to predict the experimentally resolved dimer conformation, supporting the poor agreement between simulation and experiment suggested by Fig 3. The standard Martini often predicts conformations in which peptides are parallel to each other to maximize the number of inter-peptide contacts. In cases like 1AFO, the dimers form at the wrong interface. This is evident from the positioning of the second peptide in the NMR structures (shown in green) on the other side of the first peptide (shown in yellow) for all the replicas (shown in red-white-blue). For 2K9J, there is some overlap with the simulation replicas and the NMR structure. However, this agreement does not occur at the dimerization site. Instead, the peptides are almost parallel, resulting in too many inter-peptide contacts (see also Fig 3). For 2KA1, there is again some overlap at a correct site. However, for 2HAC, Martini shows multiple conformations indicating irreversible binding upon first contact. Finally, for 2L34, the conformations are again mutually inconsistent among the replicas.

While these results are not very encouraging, we must point out that the dimerization free energies calculated earlier are not affected by the poor prediction of dimer configurations, as the dimer configurations were obtained directly from structures resolved with NMR. It must be kept in mind, though, that the crossing angle deviation, RMSD, and the deviation in the number of inter-peptide backbone contacts were compared to the NMR structure, which is not always determined in a bilayer environment.

### Performance of the scaling approaches

Having demonstrated the poor agreement between the standard Martini and experimental results, we move on to evaluate whether the issues can be corrected by scaling down the protein–protein interactions. As a first test, for the EphA1 dimer, we attempted the “W-0.6” fix, *i.e.* scaling down the interactions among the beads in contact with water. This approach was based on the promising results obtained by Stark *et al*. on water-soluble proteins [32]. This approach provided a value of −23.2 ± 0.8 kJ/mol, which is ∼50% too high compared to experimental results. Even further reduction of the interactions among the water-touching residues (W-0.8 or W-0.9) does not reduce the value of Δ*G*_DIM_ to the experimental value (see Table 1), providing concrete evidence that also the hydrophobic residues need to be scaled. Even if this strong scaling had provided good agreement with the experiment, it would have removed all chemical specificity from the model, since for example at *α* = 0.9 the most repulsive and the most attractive interactions are separated by only ∼0.4 kJ/mol (2.0 vs. 2.36 kJ/mol).

Next, we applied the uniform scaling approach (“U”) to the EphA1 dimer. We first scaled the LJ *ϵ* values using scaling factors *α* in the range of 0.1–0.9 with a spacing of 0.1 (see Eq (3)). After 250 ns of free energy (umbrella sampling) simulations per window, the obviously wrong values of *α* in each strategy were discarded, and the simulations were continued to 10 μs per window using only the most promising candidates (U-0.1 and U-0.2). Also, the number of simulated windows was limited at this point, as only the windows that were required to reach the convergence of free energy profiles were extended to 10 μs. Depending on the scaling approach and the dimer, this plateau was reached at 2.6–3.6 nm.

As shown in Table 1, the best agreement with experiment with the EphA1 dimer is obtained with the U-0.1 approach, *i.e.* when the protein–protein interactions among all protein beads are reduced by 10%. This approach gives a value of −15.2 ± 1.0 kJ/mol, which is consistent, within error margins, with the value obtained from experiment. Using stronger scaling (*α* > 0.1) leads to unstable dimerization as the minimum in the free energy profile vanishes.

Applying the U-0.1 approach to the ErbB1 dimer, we obtained a value of −15.3 ± 0.3 kJ/mol, *i.e.* the dimer is somewhat too stable in the simulation but the dimerization free energy is still sufficiently low for reversible binding that allows sampling of different dimerization interfaces (see Table 1). Meanwhile, stronger scaling did not lead to a stable dimer. This hints that a uniform approach is not sufficient, but a more careful amino-acid specific adjustment of the protein–protein interactions might be required.

Next, we verified that the U-0.1 scaling approach is gentle enough to not result in the unfolding of proteins. The S1 File (see the SI) demonstrates that protein structures are stable also with the scaling.

Having established U-0.1 as the most promising scaling approach, we evaluated its performance in predicting the structures of the spontaneously forming dimers as well as the level of oligomerization. These are tasks for which the standard Martini was shown above to be not optimal. As shown in S3 Fig, using the U-0.1 scaling, the eight ErbB1 monomers form one tetramer and two dimers, which is a clear improvement over the octamer observed for standard Martini. For EphA1, on the other hand, the performance was not improved by the U-0.1 scaling, and two tetramers were observed. It is not possible to ensure that the clustering process has reached equilibrium in our simulations. However, it is unlikely that the formed oligomers would decrease in size if the simulations were extended further. Equilibrium oligomer sizes are overestimated for both models. However, we observe several promising unbinding events with the scaled model, which suggests that the scaling takes the behavior of the system in the correct direction.

Moving on to the structures of spontaneously formed dimers, as shown in S2 Fig, the dimers form spontaneously even when the protein–protein interactions are reduced, however the formation of dimers takes more time when the U-0.1 scaling is employed. This allows the dimers to sample dimerization interfaces before a stable dimer is formed. Even one brief dissociation event is observed for the 2K9J dimer with the U-0.1 scaling.

Unfortunately, as shown in Fig 4, the agreement with experiment is not improved systematically with the U-0.1 scaling, and as with the standard Martini, all replicas fall outside the region highlighted in green. The scaled Martini model provides mutually consistent behavior among the replicas for most of the dimers (2K9J, 2KA1, and 2HAC). However, the only decent agreement with experiment is observed for 2HAC, for which all replicas show substantial overlap with the NMR structure. However, the deviations grow towards the bottom part of the structure resulting in a substantial deviation in the tilt angle (see Fig 3).

At this point, it is clear that the properties discussed here seem to be very sensitive to the scaling parameters. As an example, free energies of dimerization can change by multiple dozen kilojoules per mole upon a decrease of only 10% in the Lennard-Jones interactions. Similarly, this small decrease affects the structures of dimers quite drastically as shown in Fig 3. Based on these observations, any scaling scheme should be always adapted with care, and its validity should be evaluated by comparison with experiment.

The U-0.1 scaling should benefit studies on membrane dynamics as it prevents excessive and unrealistic clustering. It should also help proteins sample protein dimerization interfaces before actually forming a dimer. One would also assume that a uniform scaling would not affect the preference of the protein dimerization interfaces. However, the proper sampling of dimerization interfaces of membrane proteins such as GPCRs requires substantial effort and simulation time [7, 28, 35, 43]. Since such a thorough study is beyond the scope of this work, the validity of the scaling approach in predicting protein dimerization interfaces remains to be studied.

With the apparent limitations in the ability of the standard Martini model to describe protein–protein interactions, and considering that our two approaches to adjust the interaction levels also fail in predicting the correct dimerization interfaces, the obvious question is how the situation could be improved. Our results show that the reduction in protein–protein interactions required for membrane proteins to reproduce experimental dimerization free energies (10%) is much smaller than for water-soluble proteins (60% [32]). Importantly, the amino acid content in these two kinds of proteins is very different, suggesting that an amino acid specific scaling strategy, even though being very laborious and beyond the scope of this work, could provide an approach applicable to a wide range of protein types. Notably, such a technique would be especially crucial for studies involving full-length membrane proteins where ectodomains and cytosolic regions are largely exposed to interactions with water, while the TM domains are mainly in a hydrophobic environment. Currently, however, such amino acid specific scaling is also limited by the lack of quantitative experimental data for validation, covering both membrane proteins and water soluble proteins, let alone proteins with segments inside and outside the membrane.

Membrane lipids also play a key role in the oligomerization of membrane proteins. A recent study by Castillo *et. al* showed that a large hydrophobic mismatch can stabilize a dimer substantially [56]. Furthermore, the role of membrane lipids in altering the rate and the interface of receptor oligomerization were also recently demonstrated by computer simulations [7, 77]. In the free energy calculations described in this work, the role of lipids was accounted for; the membrane compositions followed those used in the respective experiments, and the correct dimerization interfaces were sampled as the conformations were derived from the dimer structure resolved by NMR. Also, the changes in Δ*G*_DIM_ due to hydrophobic mismatch [56] were smaller than the observed deviations between experiments and Martini and cannot therefore explain their poor mutual agreement. Furthermore, in our recent work [7] we observed that scaling down protein–protein interactions decreased the rate of oligomerization systematically for two different lipid compositions pointing out to a general trend that cannot be explained by the choice of lipids.

Coarse-grained simulations lack many details provided by their atomistic counterparts. Notably, in addition to van der Waals forces and steric repulsion, entropic effects and hydrogen bonding are also captured in the LJ potential in the coarse-grained scheme. Therefore, in order to resolve the nature of protein–protein and protein–lipid interactions in detail, the fine-graining of the simulated structures to atomistic resolution, followed by further simulation, is recommended. Fortunately, the recent developments in resolution transformation tools (martinize and backward [29]) provide easy access to such effective multiscale simulations. In these simulations, it is of crucial importance that the interactions are reasonably similar at the used resolutions. Otherwise efficient CG simulations might take the system to an unfavorable state—such as a tightly bound protein aggregate or a protein with its lipid binding sites covered by other proteins—from which the united-atom or all-atom simulations cannot escape.

## Conclusions

Membrane protein dimerization free energies calculated using the coarse-grained Martini model [21–23] are often much larger than the corresponding experimental values by tens of kilojoules per mole. If dimerization free energies in simulation models are too large, or even excessive, they can result in instantaneous, nonselective, and irreversible binding, thereby leading to formation of unrealistic protein oligomers or superaggregates. This further complicates the interpretation of simulation data for dimerization interfaces: if the binding between two proteins is disproportionately strong and fast, then the dimerization interface predicted by coarse-grained simulations may be wrong. Also, the aggregates resulting from excessively large dimerization free energies may give rise to unrealistically pronounced confinement effects for lipids and proteins diffusing in the membrane plane, thereby impairing the lateral dynamics of membrane systems. All these issues complicate the interpretation of experimental data with the aid of molecular dynamics simulations based on the coarse-grained Martini force field.

In this work, we attempted to improve this situation by scaling the interaction between the membrane proteins. The results based on this strategy are partly encouraging. We found that only a small reduction of approximately 10% in the LJ interactions is required to bring the dimerization free energies of RTK TM domains to the same ballpark with values obtained from FRET experiments. This signals that the Martini parametrization together with its simple combination rules gets impressively close to optimal values in a task that the Martini model was never parametrized for. Meanwhile, the small reduction in the LJ interactions did not improve agreement between the structures of spontaneously formed dimers found in simulations and those resolved from NMR, and the degree of oligomerization was still too large as compared to experiments.

Martini has also occasionally been able to predict the most favorable dimer structures [35, 43], even though their absolute binding energies are not in line with experiment. In this case, the slight and universal decrease of protein–protein interactions will likely not affect the relative affinities of the interfaces, yet it will allow the proteins to sample more conformations until they find the optimal orientation. However, this remains to be confirmed in future studies.

To conclude, our study brings about an issue in the description of protein–protein interactions in the Martini model. We consider that our work sets a promising basis for further studies to design a more accurate parametrization strategy based on, *e.g.*, amino acid specific scaling of protein–protein interactions. In the meantime, the scaled version of Martini, as discussed in this paper, can be used to improve studies on membrane dynamics and protein–lipid interactions. However, in all cases a careful comparison of simulation predictions with biochemical experiments must be performed whenever possible.

## Supporting information

S1 Table Dimer reference crossing angles. Residues used for the calculation of dimeric crossing angles. Pept-1 and Pept-2 show the two peptides consituting the dimers. The indicated residues from both peptides (numbering starting from 1) were used in the calculation; the crossing angle is defined as the angle between the principal moments of inertia of the backbone beads of the indicated residues. The angle extracted from the PDB structures is indicated by “Ang-PDB”, while that calculated in this work is labeled “Ang-Exp.”. Note that we omit the handedness and only show positive crossing angle values.
(XLSX)

S1 Fig Monomer tilt angles. Tilt angles of ErpB1 monomers with respect to the membrane normal as a function of COM–COM separation (COM stands for the centre of mass). The systematic increase of the angle, seen in the unscaled Martini model (red and black curves), is due to the strong protein–protein interaction: when the peptides are separated, they tilt in order to keep the termini at one end in contact with each other.
(PDF)

S2 Fig Peptide–peptide distances. Distance between the peptide COMs as a function of simulation time in the unbiased simulations. Data are shown for the 10 repeats done for each system, and the insets show the data for the last 20 μs in more detail.
(PDF)

S3 Fig Order of oligomerization. A) The time evolution of the average oligomer size of EphA1 and ErbB1 peptides. B) The histogram of the oligomer sizes during the last 10 μs of the simulation.
(PDF)

S1 File Stability of BtuB membrane protein with the U-0.1 scaling. Details of a simulation of a BtuB membrane protein. Stability analysis comparing the results between the normal (unscaled) Martini model and the U-0.1 scaling.
(PDF)
