# Supplementary figures and images for: Excessive aggregation of membrane proteins in the Martini model
Source: PLoS One. 2017 Nov 13;12(11):e0187936. doi: 10.1371/journal.pone.0187936 (PMC5683612; doi:10.1371/journal.pone.0187936)

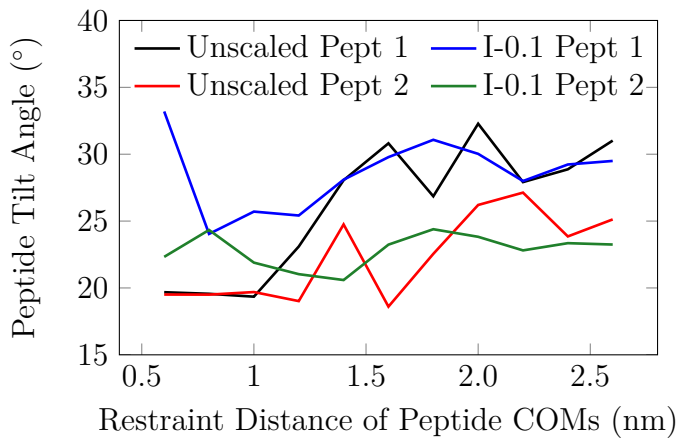

Supplement: S1 Fig — Tilt angles of ErpB1 monomers with respect to the membrane normal as a function of COM–COM separation (COM stands for the centre of mass). The systematic increase of the angle, seen in the unscaled Martini model (red and black curves), is due to the strong protein–protein interaction: when the peptides are separated, they tilt in order to keep the termini at one end in contact with each other. (PDF) [file pone.0187936.s002.pdf]

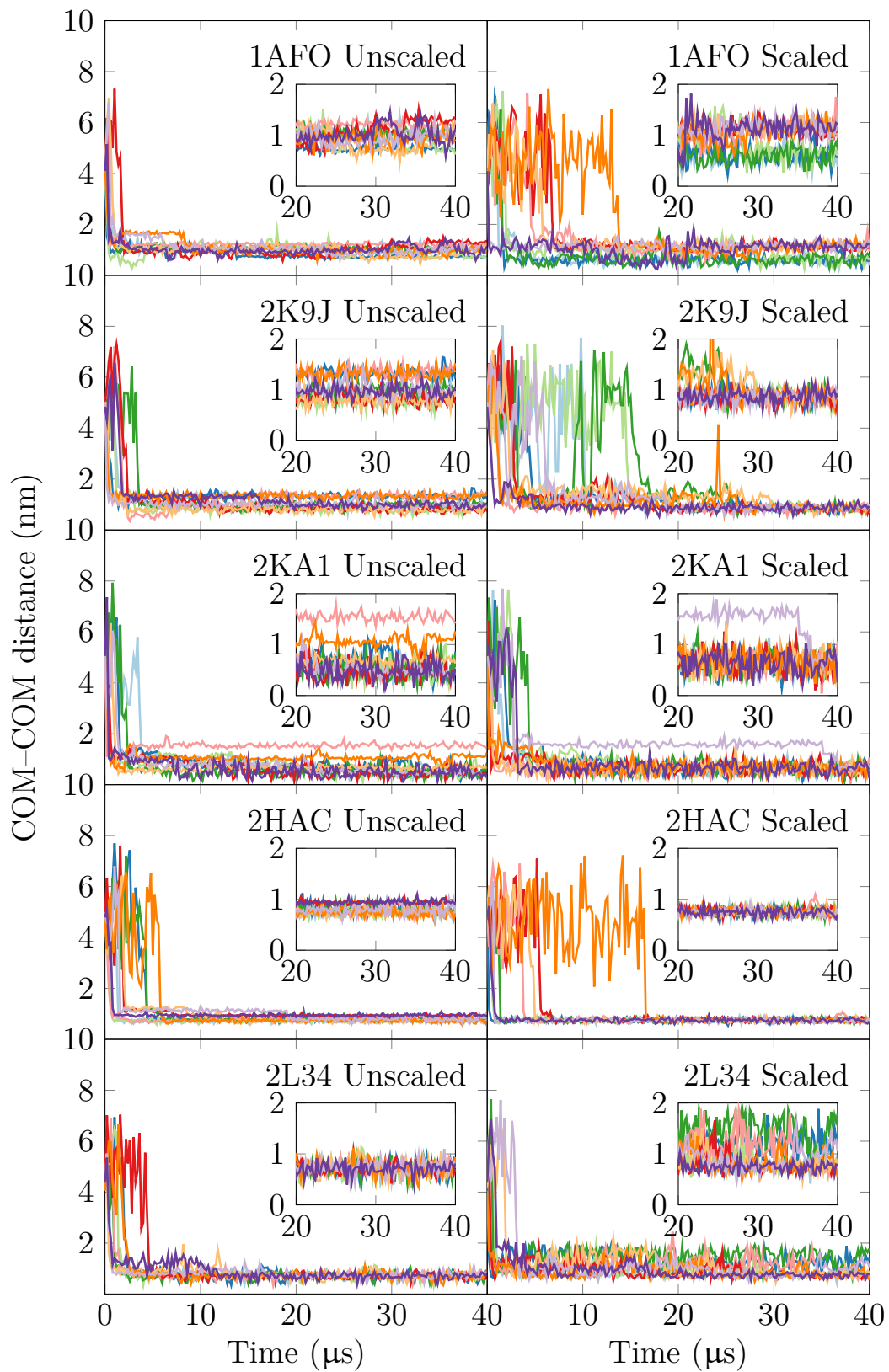

Supplement: S2 Fig — Distance between the peptide COMs as a function of simulation time in the unbiased simulations. Data are shown for the 10 repeats done for each system, and the insets show the data for the last 20 μs in more detail. (PDF) [file pone.0187936.s003.pdf]

Average cluster size

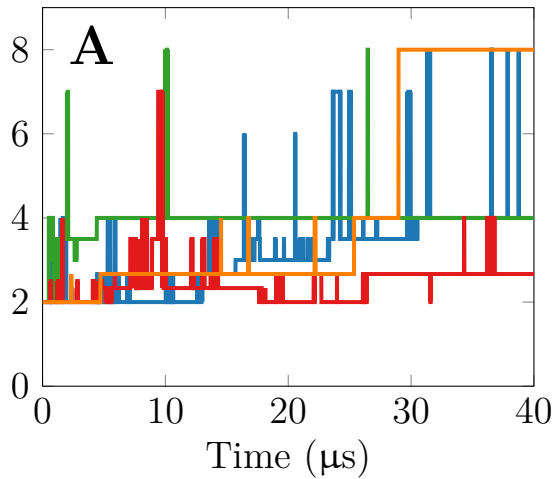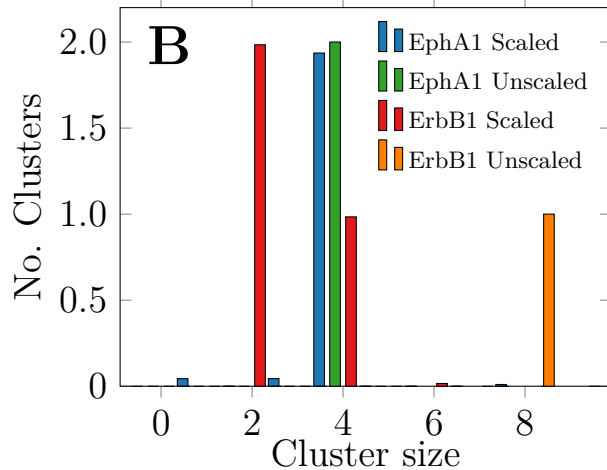

Supplement: S3 Fig — A) The time evolution of the average oligomer size of EphA1 and ErbB1 peptides. B) The histogram of the oligomer sizes during the last 10 μs of the simulation. (PDF) [file pone.0187936.s004.pdf]
