# Supplementary material for: Excessive aggregation of membrane proteins in the Martini model
Source: PLoS One. 2017 Nov 13;12(11):e0187936. doi: 10.1371/journal.pone.0187936 (PMC5683612; doi:10.1371/journal.pone.0187936)
Supplement: S1 File — Details of a simulation of a BtuB membrane protein. Stability analysis comparing the results between the normal (unscaled) Martini model and the U-0.1 scaling. (PDF) [file pone.0187936.s005.pdf]

Stability of Membrane Protein Structures

The scaling strategies explored in this work affect only the protein–protein interactions, and therefore protein–lipid and protein–water interactions as well as membrane properties remain unaltered. The secondary structure is fixed in Martini by bonded parameters. However, hydrogen bonding, van der Waals forces, and hydrophobic and electrostatic interactions define the tertiary structure of the proteins. The first three of these interactions are essentially modeled by the LJ potential in Martini, and therefore a drastic reduction of ϵ might destabilize the tertiary structure. To check the effect of the scaling of protein–protein interactions on structural protein fluctuations, we simulated the outer membrane cobalamin transporter (BtuB) [1] in a DPPC bilayer (400 lipids per leaflet) at 320 K for 1 µs using simulation parameters identical to those listed in the Methods section ("New-RF"). We considered both unscaled Martini and the I-0.1 scaling and evaluated protein stability by RMSD. These RMSD data, shown in Fig. S1, confirm that the I-0.1 strategy, especially when combined with the elastic network [2], is sufficiently gentle to avoid artificial protein unfolding.

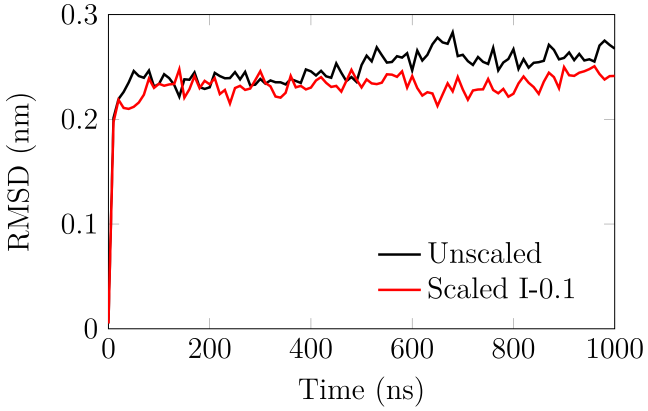

Figure S1: Root mean squared deviation (RMSD) of the BtuB protein in a DPPC membrane (see text for details). Data shown here are for the normal (unscaled) Martini model and for the I-0.1 scaling.

References

[1] David P Chimento, Arun K Mohanty, Robert J Kadner, and Michael C Wiener. Substrate-Induced Transmembrane Signaling in the Cobalamin Transporter BtuB. *Nat. Struct. Mol. Biol.*, 10(5):394–401, 2003.

[2] Xavier Periole, Marco Cavalli, Siewert-Jan Marrink, and Marco A Ceruso. Combining an Elastic Network With a Coarse-Grained Molecular Force Field: Structure, Dynamics, and Intermolecular Recognition. *J. Chem. Theory Comput.*, 5(9):2531–2543, 2009.
